# Adaptation of the Tele-Harm Reduction intervention to promote initiation and retention in buprenorphine treatment among people who inject drugs: a retrospective cohort study

**DOI:** 10.1080/07853890.2023.2182908

**Published:** 2023-03-01

**Authors:** Edward Suarez, Tyler S. Bartholomew, Marina Plesons, Katrina Ciraldo, Lily Ostrer, David P. Serota, Teresa A. Chueng, Morgan Frederick, Jason Onugha, Hansel E. Tookes

**Affiliations:** a Department of Psychiatry and Behavioral Sciences, University of Miami Miller School of Medicine, Miami, FL, USA;; b Division of Health Services Research and Policy, Department of Public Health Sciences, University of Miami Miller School of Medicine, Miami, FL, USA; c University of Miami Miller School of Medicine, Miami, FL, USA; d Department of Family and Community Medicine & Department of Obstetrics, Gynecology and Reproductive Sciences, University of Miami Miller School of Medicine, Miami, FL, USA; e Division of Infectious Diseases, Department of Medicine, University of Miami Miller School of Medicine, Miami, FL, USA; f Department of Psychiatry and Behavioral Sciences, Feinberg School of Medicine, Northwestern University, Chicago, IL, USA

**Keywords:** People who inject drugs, medications for opioid use disorder, telehealth, syringe services programs

## Abstract

**Background:** At the start of the pandemic, relaxation of buprenorphine prescribing regulations created an opportunity to create new models of medications for opioid use disorder (MOUD) delivery and care. To expand and improve access to MOUD, we adapted and implemented the *Tele-Harm Reduction (THR)* intervention; a multicomponent, telehealth-based and peer-driven intervention to promote HIV viral suppression among people who inject drugs (PWID) accessing a syringe services program (SSP). This study examined buprenorphine initiation and retention among PWID with opioid use disorder who received the adapted *THR* intervention at the IDEA Miami SSP.

**Methods:** A retrospective chart review of participants who received the *THR* intervention for MOUD was performed to examine the impact of telehealth on buprenorphine retention. Our primary outcome was three-month retention, defined as three consecutive months of buprenorphine dispensed from the pharmacy.

**Results:** A total of 109 participants received the adapted *THR* intervention. Three-month retention rate on buprenorphine was 58.7%. Seeing a provider *via* telehealth at baseline or any follow up visit (aOR = 7.53, 95% CI: [2.36, 23.98]) and participants who had received an escalating dose of buprenorphine after baseline visit (aOR = 8.09, 95% CI: [1.83, 35.87]) had a higher adjusted odds of retention at three months. Participants who self-reported or tested positive for a stimulant (methamphetamine, amphetamine, or cocaine) at baseline had a lower adjusted odds of retention on buprenorphine at three months (aOR = 0.29, 95% CI: [0.09, 0.93]).

**Conclusions:** Harm reduction settings can adapt dynamically to the needs of PWID in provision of critical lifesaving buprenorphine in a truly destigmatising approach. Our pilot suggests that an SSP may be an acceptable and feasible venue for delivery of *THR* to increase uptake of buprenorphine by PWID and promote retention in care.

**KEY MESSAGES**
- The Tele-Harm Reduction intervention can be adapted for initiating and retaining people who inject drugs with opioid use disorder on buprenorphine within a syringe services program setting
- Using telehealth was associated with increased three-month buprenorphine retention
- Baseline stimulant use was negatively associated with three-month buprenorphine retention

## 1. Introduction

Fatal overdose rates attributed to the ongoing opioid and stimulant crises continue to increase despite availability of efficacious medications for opioid use disorder (MOUD) [1]. FDA approved medications such as buprenorphine and methadone have been shown to reduce overdose mortality by over 40% [2,3] after treatment initiation. Yet, nearly 90% of people with an opioid use disorder (OUD) do not receive evidence-based treatment [4]. Accessing MOUD remains challenging with overlapping barriers at policy (health insurance coverage, Medicaid expansion, DATA waiver requirements), community (lack of certified prescribers, pharmacy issues) and individual (cost, transportation, stigma, housing) levels [5–11].

Prior to the COVID-19 pandemic, SAMHSA guidance on OUD treatment dictated a rigid and high-barrier approach to care including the Ryan Haight Act’s requirement that patients with OUD have an in-person examination with a provider before initial prescription of buprenorphine. At the start of the pandemic in March 2020, the US Drug Enforcement Agency issued an emergency order to waive this requirement [12], thus facilitating increased access to buprenorphine and opening an opportunity to develop new models of MOUD delivery and care, including prescription *via* telehealth [13]. The rise of telehealth-based opioid treatment and insurance reimbursement in the COVID era offers increased utilization of buprenorphine, comparable rates of retention, higher patient satisfaction and overall reduction in health care costs compared to office-based MOUD programs in urban and rural settings [14–18].

Syringe services programs (SSPs), long endorsed by the Centers for Disease Control and Prevention, are efficacious public health interventions declared as a key strategy to address all four pillars of the Ending the HIV Epidemic initiative in the US [19,20]. When SSPs are scaled up in collaboration with substance use treatment and antiretroviral therapy, reduction in HIV transmission among people who inject drugs (PWID) reaches nearly 50% [21]. As trusted entities among PWID, SSPs hold promise for expanding access to MOUD by offering low barrier services in locations that people with OUD frequent and trust [12,16,22–28]. Although only 20% of SSPs nationally provided one or more forms of MOUD treatment on-site in 2019 [29], there has been a significant increase in telehealth-based buprenorphine programs at SSPs in recent years [12]. However, there remains limited evidence on effective telehealth-based interventions to promote buprenorphine initiation and retention among PWID within an SSP setting [30,31].

To expand and improve access to MOUD, the IDEA Miami SSP adapted and implemented the *Tele-Harm Reduction (THR)* intervention for buprenorphine initiation and retention. *THR* is a multicomponent, telehealth-enhanced, peer-driven intervention that has shown promise for PWID living with HIV to initiate HIV care and achieve viral suppression [32]. In *THR,* telehealth is an on-demand videoconference facilitated by a peer assisting a participant interact with their physician *via* tablet at the SSP or on the mobile unit so the physician can evaluate, document, and prescribe in a remote location. Retention in care is supported by ongoing peer support and engagement with the SSP. This approach for HIV care led to 78.1% six-month viral suppression in our *THR* pilot for PWID with HIV (*n* = 35) [32], an approach now being tested in a multi-site efficacy trial. This study seeks to evaluate the feasibility of the adapted *THR* intervention and examine buprenorphine initiation and three-month retention pilot outcomes among PWID with OUD in an SSP setting.

## 2. Methods

### 2.1. Human subjects

This study was approved by the Institutional Review Board at the University of Miami (IRB # 20220658). The participants in this study were included *via* a retrospective chart review with consent waived.

### 2.2. Study setting

IDEA Miami SSP was founded in 2016 as the first legal SSP in Florida. Since inception, over 2100 PWID have enrolled in the program to access syringe services. IDEA Miami is housed within the University of Miami Miller School of Medicine in partnership with the county safety-net hospital, Jackson Health System. IDEA Miami exchanges up to 10,000 syringes weekly and currently operates three fixed and five mobile sites within Miami-Dade County. In February of 2021, the IDEA Miami SSP obtained State Opioid Response (SOR) funding and implemented a MOUD clinic named the **B**up**R**enorphine **I**nitiation and **T**reatment **E**xperience (BRITE IDEA program). Leveraging the existing infrastructure of the *THR* intervention for HIV care at the IDEA Miami SSP, the BRITE clinic immediately adapted the *THR* intervention in a pilot for low barrier buprenorphine treatment.

### 2.3. Adapted Tele-Harm Reduction intervention

*THR* was emergently adapted in iterative consultation with staff peers to facilitate low barrier MOUD for PWID with OUD. Using a community driven approach, we included people with lived OUD experience in the adaptation process for the *THR* intervention, including SSP staff peers and participants. This process is described in more detail in Tookes et al. [32]. In Component 1 of the *THR* adaptation, PWID were connected to same-day visits with a medical provider and psychologist *via* an onsite peer specialist through telehealth. In this pilot, *THR* was expanded to more broadly support PWID health according to the diverse needs of the community, meeting them where they are in a true harm reduction approach [33]. During the THR encounter, PWID would meet with one of the SSP’s bilingual physicians for evaluation and diagnosis of opioid use disorder. Recognizing PWID as the true experts in their own health, *via* shared decision-making, individualized plans for MOUD were mutually agreed upon, including choosing a specific sublingual buprenorphine dose and formulation. Physician visits were completed face-to-face at the SSP if there was a physician on site or *via* telehealth, with either the physician or participant joining remotely with videoconferencing software. Using a HIPAA compliant videoconferencing platform on a tablet in a confidential space at the fixed site SSP or on the mobile unit, peers connected participants with off-site physicians. Concurrent substance use disorders (SUDs) were also diagnosed. After the initial visit with the physician was completed, medications were e-prescribed and sent to a local pharmacy that was in close proximity to the SSP. Patients were prescribed 30 days of medication uniformly, but at times dose was escalated and the prescription was modified. Prescribing was left to physician discretion without a set protocol, with shared decision making with the patient. A third-party authorization and consent for storage of medication was obtained by the peer specialist and medications were either picked-up by SSP staff or delivered directly to the SSP for the participant to access. Number of refills were provider dependent and follow-up visits were not required to receive a refill.

In Component 2 of the *THR* intervention [32], robust wraparound support services were used, including storage of medications on site at the SSP, peer-facilitated medication deliveries, peer-facilitated telehealth follow-ups and linkage to housing to support individualized recovery trajectories. Component 2 was facilitated by two full time peers with lived substance use disorder experience whose encounters focused on managing medication, meeting basic needs and improving participant self-efficacy through motivational interviewing techniques [34]. Under the supervision of the SSP’s onsite psychologist, the peers worked alongside participants to deconstruct barriers to care, better manage co-morbidities related to mental health and increase chances of medication adherence. Many participants were either sleeping on the street or experiencing some form of unstable housing; thus, participants were offered flexible medication management protocols that included onsite storage of MOUD *via* pill lockers at the SSP. If participants were unable to independently obtain medications at the fixed site location, one of the peer specialists would find the participant on outreach and complete a medication delivery, placing the medications directly into the hands of the participant. Number of doses provided by the peer depended on participant need and was not decided at the physician level. Participants in need of treatment for co-occurring mental health disorders were connected to the SSP’s psychologist *via* telehealth or in-person for mental health assessment and treatment.

### 2.4. Data collection and measures

All participants who accessed care through BRITE *via* our drop-in telehealth or in-person clinical visits between 1 February 2021 through 30 May 2022 and were prescribed buprenorphine were included in a retrospective chart review. The Electronic Health Record (EHR) was utilized to abstract data from physician notes, psychologist notes and laboratory values. The procedure consisted of recording initial and follow up appointment dates with the physician, prescription order records (including doses and dates) and UDS results. Types of substances used by the participant were identified within physician notes, which included participant self-report or UDS results, depending on the modality of the encounter. The UDS tested for cocaine, methamphetamine, amphetamine, 3,4-methylenedioxy-methamphetamine (MDMA), cannabis, methadone, buprenorphine, benzodiazepines, morphine, oxycodone, phencyclidine and ethyl glucuronide. Many BRITE participants self-reported injecting ‘molly’, but recent findings suggests this substance is likely a synthetic cathinone [35]. UDS were used to facilitate diagnosis and management and were not used punitively. UDS allowed physicians to assess response to treatment and need for intensification of support services and confirm the participant was taking the buprenorphine.

Florida’s Prescription Drug Monitoring Program (PDMP) was utilized to collect data on the dates when buprenorphine prescriptions were picked up at the pharmacy by either the participant or SSP staff and to confirm the doses of these prescriptions. SSP staff only called refills into the pharmacy at the request of participants. Only buprenorphine prescribed by SSP physicians were included. MOUD initiation was defined as first collection of buprenorphine from the pharmacy. Measurement of buprenorphine dosage prescribed was tracked utilizing the PDMP to identify trends in escalation. Daily dosages of buprenorphine were 8 mg, 16 mg, 24 mg or 32 mg per day, and escalation was defined as prescription of a higher dose after the baseline visit.

Sociodemographic data and engagement in telehealth services were abstracted from clinical notes, the BRITE clinic enrollment form and the IDEA SSP administrative database. Engagement with a provider *via* telehealth was defined as having at least one MD visit *via* telehealth at baseline or follow-up visit (yes/no). HIV and HCV status were abstracted by cross referencing physician notes and laboratory values in the EHR with the SSP administrative database. All abstracted data were de-identified and recorded in REDCap for data management and the trained data abstracters met on a weekly basis to enhance data quality [36].

### 2.5. Primary outcome

The primary outcome of retention was defined as three consecutive months of buprenorphine prescriptions picked up at the pharmacy in the 90 days post *THR* enrollment as indicated on the Florida PDMP.

### 2.6. Statistical analysis

Baseline characteristics for the total sample and by three-month retention status (retained or not retained) are reported. Descriptive analyses comparing those retained and not retained at three months were conducted using the Pearson’s chi-square test or Fisher’s exact test for categorical variables. To examine the association between telehealth-based care visits and three-month retention, a multivariable logistic regression model was constructed that controlled for age, biological sex, and race/ethnicity. Variables in bivariate analyses with *p* values <.15 and theoretically hypothesized to impact our primary outcome were included as covariates in the adjusted model [30] to be more inclusive of potential confounders. Variables that were included were: insurance status at enrollment, housing status at enrollment, escalating the dose of buprenorphine after enrollment, baseline stimulant use and telehealth-based visits to examine their association with three-month retention. Adjusted odds ratios (aOR) and corresponding 95% confidence intervals were reported. All analyses were conducted using SAS software, Version 9.4 (SAS Institute Inc., Cary, NC, USA) and significance was set at an alpha of 0.05.

## 3. Results

A total of 109 participants who received the adapted *THR* intervention were included in the final analytic sample. The majority of participants were males (75.9%) and uninsured (78.7%). Racial demographics included non-Hispanic White (49.5%), Hispanic (35.8%) and non-Hispanic Black (14.7%). Most participants reported being either unstably housed or rough sleeping (70.4%) and were current participants in the IDEA Miami SSP (61.3%). The median age was 38 years old (IQR: 30–46). Majority of participants either self-reported or tested positive on UDS for fentanyl (71.6%) or a stimulant (53.2%) during the enrollment period. At the majority of first physician visits, providers prescribed a 30-day supply at a 16 mg dose per day (67.9%). The overall three-month retention rate on buprenorphine was 58.7% (Table 1).

**Table 1.** Descriptive statistics of participants in the adapted Tele-Harm Reduction pilot (*n* = 109).

Characteristic	Total sample
Age (median, IQR)	38 (30–46)
Biological sex (n,%)	
Male	82 (75.9)
Female	26 (24.1)
Race/ethnicity (n,%)	
Non-Hispanic White	54 (49.5)
Non-Hispanic Black	16 (14.7)
Hispanic	39 (35.8)
Insurance status at enrolment (n,%)	
Insured	12 (11.1)
Uninsured	85 (78.7)
Underinsured^a^	11 (10.2)
Housing status at enrolment (n,%)	
Stably housed	37 (34.3)
Unstably housed (in shelter)	39 (36.1)
Rough sleeping (street)^b^	32 (29.6)
IDEA SSP participant (n,%)	
Yes	65 (61.3)
No	41 (38.7)
Substance use at baseline	
Methamphetamine/amphetamine	22 (20.2)
Cocaine	48 (44.0)
Buprenorphine	32 (29.4)
MDMA	11 (10.1)
‘Molly’	7 (6.4)
Fentanyl	78 (71.6)
Heroin	15 (13.8)
Marijuana	36 (33.0)
Alcohol	16 (14.7)
Benzodiazepine	19 (17.4)
HIV status (*n* = 85) (n,%)	
Positive	5 (5.9)
Negative	80 (94.1)
HCV Ab status (*n* = 89) (n,%)	
Positive	35 (39.3)
Negative	54 (60.7)
Starting buprenorphine dose (n,%)	
8 mg	6 (5.7)
16 mg	72 (67.9)
24 mg	23 (21.7)
32 mg	1 (1.9)
Retained at three months (n,%)	
Yes	64 (58.7)
No	45 (41.3)

^a^Insurance did not cover or did not approve prior authorisation for buprenorphine.

^b^Rough sleeping was defined as anyone who was living/sleeping on the streets or in public areas.

On bivariate analyses, participants who engaged with a provider *via* telehealth at least once (75.0% vs. 26.3%, *p* < .01) and who received an escalating dose of buprenorphine at a follow up visit (86.7% vs. 48.1%, *p* < .01) were significantly more likely to be retained at three months. In addition, participants who self-reported or tested positive for a stimulant on UDS at baseline were significantly less likely to be retained on buprenorphine at three months (46.6% vs. 72.6%, *p* < .01). There were no significant differences in three-month retention across race/ethnicity and housing status categories (Table 2).

**Table 2.** Demographic and tele-harm reduction component comparisons between those retained and not retained in buprenorphine treatment at three months.

Characteristic	Retained at three months (*n* = 64)	Not retained at three months (*n* = 45)	*p* Value
Age (n,%)			.91
< =29 years old	12 (60.0)	8 (40.0)	
30–39 years old	22 (56.4)	17 (43.6)	
40–59 years old	27 (58.7)	19 (41.3)	
> =60	3 (75.0)	1 (25.0)	
Biological sex (n,%)			.32
Male	50 (61.0)	32 (39.0)	
Female	13 (50.0)	13 (50.0)	
Race/ethnicity (n,%)			.61
Non-Hispanic White	34 (63.0)	20 (37.0)	
Non-Hispanic Black	8 (50.0)	8 (50.0)	
Hispanic	22 (56.4)	17 (43.6)	
Insurance status at enrolment (n,%)			.03
Insured	7 (63.6)	4 (36.4)	
Uninsured	42 (51.9)	39 (48.2)	
Underinsured	14 (87.5)	2 (12.5)	
Housing status at enrolment (n,%)			.13
Stably housed	20 (54.1)	17 (46.0)	
Unstably housed (in shelter)	28 (71.8)	11 (28.2)	
Rough sleeping (street)	16 (50.0)	16 (50.0)	
IDEA SSP participant (n,%)	40 (61.5)	25 (38.5)	.47
Stimulant use at baseline	27 (46.6)	31 (53.5)	<.01
Saw provider *via* Telehealth in first three months (n,%)	54 (75.0)	18 (25.0)	<.01
Escalated buprenorphine dose post baseline (n,%)	26 (86.7)	4 (13.8)	<.01

In the multivariable logistic regression model, after adjusting for age, sex, race/ethnicity, insurance status at enrollment and housing status at enrollment, seeing a provider *via* telehealth at any follow up visit had a higher adjusted odds of retention at three months (aOR = 7.53, 95% CI: [2.36, 23.98]). In addition, participants who received an escalating dose of buprenorphine after baseline visit had a higher adjusted odds of retention at three months (aOR = 8.09, 95% CI: [1.83, 35.87]). Alternatively, participants who self-reported or tested positive for a stimulant (methamphetamine, amphetamine, or cocaine) had a lower adjusted odds of retention on buprenorphine at three months (aOR = 0.29, 95% CI: [0.09, 0.93]) (Table 3).

**Table 3.** Adjusted logistic regression model assessing the impact of Tele-Harm Reduction for buprenorphine treatment on three-month retention.

Characteristic	aOR	95% CI
Age	0.99	0.95, 1.05
Biological sex		
Male	2.45	0.60, 9.99
Female	Ref	Ref
Race/ethnicity		
Non-Hispanic Black	0.48	0.09, 2.47
Hispanic	0.84	0.23, 3.04
Non-Hispanic White	Ref	Ref
Insurance status at enrolment		
Uninsured	0.39	0.07, 2.09
Underinsured	4.71	0.37, 59.69
Insured	Ref	Ref
Housing status at enrolment		
Unstably Housed (in shelter)	2.43	0.60, 9.83
Rough sleeping (street)	1.33	0.28, 6.34
Stably Housed	Ref	Ref
Stimulant use at baseline		
Yes	**0.29**	**0.09, 0.93**
No	Ref	Ref
Escalated buprenorphine dose post baseline		
Yes	**8.09**	**1.83, 35.87**
No	Ref	Ref
Saw provider *via* Telehealth in first three months		
Yes	**7.53**	**2.36, 23.98**
No	Ref	Ref

*Note*. **Bolded** represents a *p* value <.05.

## 4. Discussion

In this study we showed that adaptation of a telehealth-enhanced harm reduction intervention for PWID was effective for initiation and retention on buprenorphine treatment. Despite a population burdened by numerous negative social determinants of health – unstable housing, uninsured status, racial/ethnic minority status – we achieved retention (58.7%) at 90-days comparable to more established office-based programs [37]. The program successfully initiated 109 patients on buprenorphine over 16 months, most of whom had no other access to therapy within the underserved local SUD treatment environment. These data highlight the critical role of SSPs and other harm reduction organizations as an access point to life-saving medical interventions for vulnerable and stigmatized populations, who are often avoidant of traditional healthcare institutions. While low-barrier access to free buprenorphine is an important component of the program, the additional peer-led support services, medication management, patient navigation and assistance with housing were also feasible in the context of this adapted *THR* intervention. In *THR*, we propose a new model of SSP-based MOUD provision that goes beyond simply using technology to prescribe buprenorphine in an innovative setting. *THR* addresses barriers to care including but not limited to lack of insurance, lack of identification, and limited pharmacy inventory [27,38].

Funding from Florida’s SAMHSA recipient, the Florida Department of Children and Families, enabled the establishment of a truly low barrier THR model. First, the SOR grant provided buprenorphine at no cost to uninsured participants, paid co-pays for insured participants and covered the full cost for participants whose insurance rejected the prior authorization. Second, the SOR grant did not require a typical buprenorphine treatment agreement, so participants were not discharged for using other substances while part of the THR pilot. In fact, our program sought to treat co-occurring SUDs with available pharmacotherapies [39–42]. This was particularly important given that drug use among the THR participants often included concurrent SUDs, with prominent co-administration of stimulants (cocaine and methamphetamine), which has been previously reported on among our IDEA Miami participants [43]. Studies have shown that stimulant use can lead to poor OUD treatment retention [44,45]; however, our THR pilot had significant success given the tremendous psychosocial and structural burden faced by our participants [46,47]. Finally, participants had flexibility with appointments and access to their medication in an authentic, on-demand, harm reduction approach. Critically, the IDEA Miami SSP peers picked up medications from the contracted pharmacy to overcome the barrier of lack of identification faced by many participants. We have previously shown that less than 30% of pharmacies in Miami-Dade County stock buprenorphine and most are unwilling to order it for a patient with a prescription [38]. The THR intervention team deftly navigated these deficiencies in our community. A key component of THR was use of the SSP’s on-site pill lockers to store medications for weekly pickup or delivery facilitated by the peer, allowing for both ongoing wellness checks and prevention of loss or theft of buprenorphine, especially given the high number of participants experiencing homelessness (65.7%).

Pursuit of racial equity has always been a top priority of IDEA as a result of the historic leadership of Black, Indigenous and people of color (BIPOC) individuals in the harm reduction movement [48]. Given the stark racial disparities in access to buprenorphine despite an escalating overdose crisis in the Black community [49–58], the IDEA Miami SSP has actively sought to increase access to buprenorphine through low barrier strategies. It has previously shown that Black PWID were more likely to access the SSP through mobile outreach; thus, the next phase of BRITE will aim to more fully leverage the SSP’s mobile unit in delivery of the THR intervention, specifically for low barrier buprenorphine in historically Black communities [59]. Black and non-Black participants had similar rates of retention in the present study, likely reflecting THR’s attention to structural racism experienced by Black participants, a strategy that can improve equity. However, it is possible that the study was underpowered to detect racial disparities. Another strategy for increasing access to low barrier MOUD and promoting health equity would be expanded access to methadone delivered *via* mobile units [60]. The stringent level of regulation of Opioid Treatment Programs by SAMHSA makes this approach a challenge for non-traditional healthcare venues such as SSPs, which in their authenticity to a harm reduction approach truly meet people where they are with dynamic adaptation in an inherently informal environment [61,62].

Centered on PWID from design through implementation, THR has an auspicious future as an adaptable intervention that sets aside the traditional, highly regulated and highly stigmatizing healthcare system to bring quality healthcare services to PWID that *they* prioritize [32]. A more comprehensive form of *THR* would aim to encompass HIV care and prevention (PrEP), MOUD, HCV treatment, and treatment of skin and soft tissue infections all in one integrated delivery model. Optimization of the intervention could include adding evidence-based tools such as contingency management to treat co-occurring stimulant use disorders [63]. The BRITE program is supervised by a clinical psychologist, assuring the *THR* intervention addresses PWID mental health and physical wellbeing. Provision of low barrier buprenorphine in this study has not only been shown to be feasible and acceptable, but the pilot outcomes suggest that this model should be tested in a randomized controlled trial to evaluate efficacy. The significant association of telehealth physician visits with increased retention supports the hypothesis that peer-facilitated access to compassionate care can have tremendous impact on retention. Furthermore, the finding that escalation of buprenorphine dose was associated with increased odds of retention is consistent with recent reports indicating the need for high dose buprenorphine in the fentanyl era [64–69].

One major challenge in this pilot study was defining retention. In traditional office-based settings providing buprenorphine, efforts to quantify retention have been complicated by diversity in how it is measured and reported [37,70–81]. There is a wide range of retention rates for buprenorphine in office-based settings, from 20 to 83% in randomized control trials and observational studies [37]. The only two implementation adaptations found to have a significant impact on retention were use of higher doses of buprenorphine and initiation of treatment while hospitalized or within criminal justice settings prior to outpatient treatment programs [70,72,79,82]. Neither supervision of medication consumption nor integration of medical, psychiatric, or social services nor information-technology approaches were found to improve retention [70,72,73]. The evidence with regard to psychosocial and behavioral interventions was mixed, with some reviews finding no improvement in retention and others finding some degree of improvement, primarily for contingency management [70,72,74,77]. In fealty to its low barrier approach, the IDEA Miami SSP did not require mutual support organization attendance, directly observed therapy, or psychiatric counseling for participation in this pilot. This study arrived at the relatively stringent definition of three consecutive months of buprenorphine prescription fills to define retention. An efficacy trial of *THR* for low barrier buprenorphine might consider a biologic outcome such as norbuprenorphine on urine drug screen.

While many papers have been published on the promise and feasibility of delivering MOUD onsite at SSPs, only a few specifically examine retention in care. An evaluation from Philadelphia found that the percentage of patients retained in care at 3, 6, 9 and 12 months was 77%, 65%, 59% and 56%, respectively, while the percentage with a positive opiates screen was 19%, 13%, 17% and 16%, respectively [83]. Another evaluation from Washington, DC found comparable rates of retention, with 82%, 65% and 59% of patients retained in care at 1, 6 and 12 months, respectively [84]. A third evaluation from New York found lower rates of retention, with 62%, 43% and 31% of patients retained in care at 1, 3 and 6 months, respectively [28]. A fourth evaluation from Seattle found that patients were prescribed buprenorphine for a median of 26% of the 180 days following enrollment [85]. The findings from the present study, with 58.7% of participants retained at care after three months, fall within the ranges in retention in care identified by the other literature, although comparison is complicated by the different definitions of retention used and the effects of the COVID pandemic.

There were limitations to this study. First, this study followed a retrospective observational design with no comparator group which limited the ability to estimate an intervention effect. Second, as THR was implemented emergently in response to the COVID pandemic, data collection systems were not optimized. Thus, information on the frequency of peer-facilitated medication deliveries was not systematically collected during our pilot phase. However, typically in THR, peer-facilitated medication deliveries occur weekly. Likewise, as a telehealth intervention in emergency response, many baseline UDS were not collected, leading to the inclusion of self-reported substance use from the physician notes. However, the SSP has trusted staff and clinicians, increasing the likelihood of accurate self-report from participants, as has been shown extensively in SUD research [86]. Third, although this study controlled for several covariates in its adjusted model, there may have been unmeasured confounders that could have biased the study results. Fourth, the definition of three-month retention was strict and may not truly represent the retention rate of the program’s participants. We are unable to confirm that prescriptions picked up by SSP staff were received by participants, but this level of data collection could be inserted into a rigorous clinical trial to test the efficacy of the *THR* intervention. While UDS was not required in this pilot, a biological outcome such as norbuprenorphine on UDS across a longer follow-up could further strengthen the retention outcome in a future efficacy trial. Finally, this pilot study was carried out in a single site, urban SSP within an academic-medical institution, thus limiting the generalizability to other SSPs in differing geographic and operational settings.

## 5. Conclusion

At three months, more than half of the IDEA Miami SSP’s *THR* participants were retained in OUD treatment, illustrating the ability of harm reduction settings, a less formal healthcare setting, to adapt dynamically to the needs of PWID in provision of lifesaving buprenorphine in a truly destigmatizing environment. In pursuit of racial equity, it will be critical to utilize diverse implementation strategies, such as mobile units, to meet PWID of color where they are. Overall, this study suggests that an SSP may be an acceptable venue for delivery of the tele-harm reduction intervention to increase uptake of buprenorphine by PWID and promote retention in care. Future research should more rigorously examine the efficacy of the THR intervention for buprenorphine initiation and retention.

## Data Availability

Data are not publicly available but may be accessed upon request.

## References

[1] CDC. Understanding the epidemic. Drug overdose; 2021. Available from: https://www.cdc.gov/drugoverdose/epidemic/index.html

[2] Larochelle MR, Bernson D, Land T, et al. Medication for opioid use disorder after nonfatal opioid overdose and association with mortality: a cohort study. Ann Intern Med. 2018;169(3):137–145. 29913516 10.7326/M17-3107 PMC6387681

[3] SAHMSA. Medication assisted treatment: buprenorphine; 2022. Available from: https://www.samhsa.gov/medication-assisted-treatment/medications-counseling-related-conditions/buprenorphine#:∼:text=Buprenorphine%20is%20an%20opioid%20partial,buprenorphine%20is%20safe%20and%20effective

[4] Krawczyk N, Rivera BD, Jent V, et al. Has the treatment gap for opioid use disorder narrowed in the U.S.?: a yearly assessment from 2010 to 2019. Int J Drug Policy. 2022;110:103786. 35934583 10.1016/j.drugpo.2022.103786 PMC10976290

[5] Beetham T, Saloner B, Wakeman SE, et al. Access to office-based buprenorphine treatment in areas with high rates of opioid-related mortality: an audit study. Ann Intern Med. 2019;171(1):1–9. 31158849 10.7326/M18-3457 PMC7164610

[6] Yager A. Connecting the dots; 2022. Available from: https://www.floridahealthjustice.org/connecting-the-dots.html

[7] Jacobson N, Horst J, Wilcox-Warren L, et al. Organizational facilitators and barriers to medication for opioid use disorder capacity expansion and use. J Behav Health Serv Res. 2020;47(4):439–448. 32347426 10.1007/s11414-020-09706-4 PMC7578054

[8] Kazerouni NJ, Irwin AN, Levander XA, et al. Pharmacy-related buprenorphine access barriers: an audit of pharmacies in counties with a high opioid overdose burden. Drug Alcohol Depend. 2021;224:108729. 33932744 10.1016/j.drugalcdep.2021.108729

[9] Cooper HLF, Cloud DH, Young AM, et al. When prescribing isn’t enough-pharmacy-level barriers to buprenorphine access. N Engl J Med. 2020;383(8):703–705. 32813945 10.1056/NEJMp2002908

[10] Frost T, Deutsch S, Brown S, et al. “We’ll be able to take care of ourselves” - a qualitative study of client attitudes toward implementing buprenorphine treatment at syringe services programs. Subst Abus. 2021;42(4):983–989. 33759722 10.1080/08897077.2021.1901173 PMC10112278

[11] Textor L, Ventricelli D, Aronowitz SV. Red flags’ and ‘red tape’: telehealth and pharmacy-level barriers to buprenorphine in the United States. Int J Drug Policy. 2022;105:103703. 35561484 10.1016/j.drugpo.2022.103703 PMC10962060

[12] Lambdin BH, Bluthenthal RN, Tookes HE, et al. Buprenorphine implementation at syringe service programs following waiver of the Ryan Haight act in the United States. Drug Alcohol Depend. 2022;237:109504. 35688052 10.1016/j.drugalcdep.2022.109504 PMC10878423

[13] Wang L, Weiss J, Ryan EB, et al. Telemedicine increases access to buprenorphine initiation during the COVID-19 pandemic. J Subst Abuse Treat. 2021;124:108272. 33771276 10.1016/j.jsat.2020.108272 PMC7833481

[14] Tofighi B, McNeely J, Walzer D, et al. A telemedicine buprenorphine clinic to serve New York City: initial evaluation of the NYC public hospital system’s initiative to expand treatment access during the COVID-19 pandemic. J Addict Med. 2022;16(1):e40–e43. 33560696 10.1097/ADM.0000000000000809 PMC8339146

[15] Weintraub E, Seneviratne C, Anane J, et al. Mobile telemedicine for buprenorphine treatment in rural populations with opioid use disorder. JAMA Netw Open. 2021;4(8):e2118487. 34448869 10.1001/jamanetworkopen.2021.18487 PMC8397932

[16] Guillen AG, et al. Utilization of telehealth solutions for patients with opioid use disorder using buprenorphine: a scoping review. Telemed J E Health. 2022;28(6):761–767. 34714172 10.1089/tmj.2021.0308

[17] Mahmoud H, Naal H, Whaibeh E, et al. Telehealth-based delivery of medication-assisted treatment for opioid use disorder: a critical review of recent developments. Curr Psychiatry Rep. 2022;24(9):375–386. 35895282 10.1007/s11920-022-01346-z PMC9326140

[18] Chan B, Bougatsos C, Priest KC, et al. Opioid treatment programs, telemedicine and COVID-19: a scoping review. Subst Abus. 2022;43(1):539–546. 34520702 10.1080/08897077.2021.1967836 PMC11970435

[19] Nassau T, Al-Tayyib A, Robinson WT, et al. The impact of syringe services program policy on risk behaviors among persons who inject drugs in 3 US cities, 2005-2015. Public Health Rep. 2020;135(1_suppl):138S–148S. 32735193 10.1177/0033354920930137 PMC7407040

[20] Javed Z, Burk K, Facente S, et al. Syringe Services Programs: A Technical Package of Effective Strategies and Approaches for Planning, Design, and Implementation. Atlanta, GA: US Department of Health and Human Services, National Center for HIV/AIDS, Viral Hepatitis, STD and TB Prevention, Centers for Disease, Control and Prevention; 2020.

[21] Broz D, Carnes N, Chapin-Bardales J, et al. Syringe services programs’ role in ending the HIV epidemic in the U.S.: why we cannot do it without them. Am J Prev Med. 2021;61(5 Suppl 1):S118–S129. 34686281 10.1016/j.amepre.2021.05.044 PMC11334402

[22] Chatterjee A, Obando A, Strickland E, et al. Shelter-Based opioid treatment: increasing access to addiction treatment in a family shelter. Am J Public Health. 2017;107(7):1092–1094. 28520496 10.2105/AJPH.2017.303786 PMC5463216

[23] Doorley SL, Ho CJ, Echeverria E, et al. Buprenorphine shared medical appointments for the treatment of opioid dependence in a homeless clinic. Subst Abus. 2017;38(1):26–30. 27897918 10.1080/08897077.2016.1264535 PMC5592108

[24] Carter J, Zevin B, Lum PJ. Low barrier buprenorphine treatment for persons experiencing homelessness and injecting heroin in San Francisco. Addict Sci Clin Pract. 2019;14(1):20. 31060600 10.1186/s13722-019-0149-1 PMC6501460

[25] Krawczyk N, Buresh M, Gordon MS, et al. Expanding low-threshold buprenorphine to justice-involved individuals through mobile treatment: addressing a critical care gap. J Subst Abuse Treat. 2019;103:1–8. 31229187 10.1016/j.jsat.2019.05.002 PMC6612429

[26] O'Gurek DT, Jatres J, Gibbs J, et al. Expanding buprenorphine treatment to people experiencing homelessness through a mobile, multidisciplinary program in an urban, underserved setting. J Subst Abuse Treat. 2021;127:108342. 34134882 10.1016/j.jsat.2021.108342

[27] Castillo M, Conte B, Hinkes S, et al. Implementation of a medical student-run telemedicine program for medications for opioid use disorder during the COVID-19 pandemic. Harm Reduct J. 2020;17(1):1–6. 33203460 10.1186/s12954-020-00438-4 PMC7671179

[28] Jakubowski A, Norton BL, Hayes BT, et al. Low-threshold buprenorphine treatment in a syringe services program: program description and outcomes. J Addict Med. 2022;16(4):447–453. 34775441 10.1097/ADM.0000000000000934 PMC9095762

[29] Behrends CN, Lu X, Corry GJ, et al. Harm reduction and health services provided by syringe services programs in 2019 and subsequent impact of COVID-19 on services in 2020. Drug Alcohol Depend. 2022;232:109323. 35124386 10.1016/j.drugalcdep.2022.109323 PMC8772135

[30] Ward KM, Scheim A, Wang J, et al. Impact of reduced restrictions on buprenorphine prescribing during COVID-19 among patients in a community-based treatment program. Drug Alcohol Depend Rep. 2022;3:100055. 35497489 10.1016/j.dadr.2022.100055 PMC9040407

[31] Lambdin BH, Kan D, Kral AH. Improving equity and access to buprenorphine treatment through telemedicine at syringe services programs. Subst Abuse Treat Prev Policy. 2022;17(1):1–5. 35841036 10.1186/s13011-022-00483-1 PMC9283820

[32] Tookes HE, Bartholomew TS, Suarez E, et al. Acceptability, feasibility, and pilot results of the tele-harm reduction intervention for rapid initiation of antiretrovirals among people who inject drugs. Drug Alcohol Depend. 2021;229(Pt A):109124. 34781096 10.1016/j.drugalcdep.2021.109124 PMC9102418

[33] Myers JE, Braunstein, SL, Xia Q, et al. (2018, June). Redefining prevention and care: a status-neutral approach to HIV. In *open forum infectious diseases* (Vol. 5, No. 6, p. ofy097). US: Oxford University Press. 10.1093/ofid/ofy097 PMC6016418 29977957

[34] Artenie AA, Fortier E, Sylvestre M-P, et al. Socioeconomic stability is associated with lower injection frequency among people with distinct trajectories of injection drug use. Int J Drug Policy. 2021;94:103205. 33839598 10.1016/j.drugpo.2021.103205

[35] Gladden RM, Chavez-Gray V, O’Donnell J, et al. Notes from the field: overdose deaths involving eutylone (psychoactive bath salts)—United States, 2020. MMWR Morb Mortal Wkly Rep. 2022;71(32):1032–1034. 35951491 10.15585/mmwr.mm7132a3 PMC9400538

[36] Harris PA, Taylor R, Thielke R, et al. Research electronic data capture (REDCap)—a metadata-driven methodology and workflow process for providing translational research informatics support. J Biomed Inform. 2009;42(2):377–381. 18929686 10.1016/j.jbi.2008.08.010 PMC2700030

[37] Klimas J, Hamilton M-A, Gorfinkel L, et al. Retention in opioid agonist treatment: a rapid review and meta-analysis comparing observational studies and randomized controlled trials. Syst Rev. 2021;10(1):216. 34362464 10.1186/s13643-021-01764-9 PMC8348786

[38] Syros A, Rodriguez MG, Rennick AC, et al. Availability of medications for opioid use disorder in outpatient and inpatient pharmacies in South Florida: a secret shopper survey. Addict Sci Clin Pract. 2022;17(1):1–9. 36401298 10.1186/s13722-022-00346-x PMC9673453

[39] Coffin PO, Santos G-M, Hern J, et al. Effects of mirtazapine for methamphetamine use disorder among cisgender men and transgender women who have sex with men: a placebo-controlled randomized clinical trial. JAMA Psychiatry. 2020;77(3):246–255. 31825466 10.1001/jamapsychiatry.2019.3655 PMC6990973

[40] Prince V, Bowling KC. Topiramate in the treatment of cocaine use disorder. Am J Health Syst Pharm. 2018;75(1):e13–e22. 29273608 10.2146/ajhp160542

[41] Tardelli VS, Bisaga A, Arcadepani FB, et al. Prescription psychostimulants for the treatment of stimulant use disorder: a systematic review and meta-analysis. Psychopharmacology. 2020;237(8):2233–2255. 32601988 10.1007/s00213-020-05563-3

[42] Trivedi MH, Walker R, Ling W, et al. Bupropion and naltrexone in methamphetamine use disorder. N Engl J Med. 2021;384(2):140–153. 33497547 10.1056/NEJMoa2020214 PMC8111570

[43] Bartholomew TS, Tookes HE, Bullock C, et al. Examining risk behavior and syringe coverage among people who inject drugs accessing a syringe services program: a latent class analysis. Int J Drug Policy. 2020;78:102716. 32146348 10.1016/j.drugpo.2020.102716 PMC7302981

[44] Samples H, Williams AR, Olfson M, et al. Risk factors for discontinuation of buprenorphine treatment for opioid use disorders in a multi-state sample of Medicaid enrollees. J Subst Abuse Treat. 2018;95:9–17. 30352671 10.1016/j.jsat.2018.09.001 PMC6354252

[45] Tsui JI, Mayfield J, Speaker EC, et al. Association between methamphetamine use and retention among patients with opioid use disorders treated with buprenorphine. J Subst Abuse Treat. 2020;109:80–85. 31810594 10.1016/j.jsat.2019.10.005

[46] Shiau S, Krause KD, Valera P, et al. The burden of COVID-19 in people living with HIV: a syndemic perspective. AIDS Behav. 2020;24(8):2244–2249. 32303925 10.1007/s10461-020-02871-9 PMC7165075

[47] Singer M. Introduction to syndemics: a critical systems approach to public and community health. John Wiley & Sons. 2009: Vol. 1, pp. 1–304.

[48] Hughes M, Suhail-Sindhu S, Namirembe S, et al. The crucial role of black, latinx, and indigenous leadership in harm reduction and addiction treatment. Am J Public Health. 2022;112(S2):S136–S139. 35349317 10.2105/AJPH.2022.306807 PMC8965189

[49] Andraka-Christou B. Addressing racial and ethnic disparities in the use of medications for opioid use disorder. Health Aff. 2021;40(6):920–927. 10.1377/hlthaff.2020.02261 34097509

[50] Goedel WC, Shapiro A, Cerdá M, et al. Association of racial/ethnic segregation with treatment capacity for opioid use disorder in counties in the United States. JAMA Netw Open. 2020;3(4):e203711. 32320038 10.1001/jamanetworkopen.2020.3711 PMC7177200

[51] Hansen H, Siegel C, Wanderling J, et al. Buprenorphine and methadone treatment for opioid dependence by income, ethnicity and race of neighborhoods in New York city. Drug Alcohol Depend. 2016;164:14–21. 27179822 10.1016/j.drugalcdep.2016.03.028 PMC5539992

[52] Hansen HB, Siegel CE, Case BG, et al. Variation in use of buprenorphine and methadone treatment by racial, ethnic, and income characteristics of residential social areas in New York City. J Behav Health Serv Res. 2013;40(3):367–377. 23702611 10.1007/s11414-013-9341-3 PMC3818282

[53] James K, Jordan A. The opioid crisis in black communities. J Law Med Ethics. 2018;46(2):404–421. 30146996 10.1177/1073110518782949

[54] Kilaru AS, Xiong A, Lowenstein M, et al. Incidence of treatment for opioid use disorder following nonfatal overdose in commercially insured patients. JAMA Netw Open. 2020;3(5):e205852. 32459355 10.1001/jamanetworkopen.2020.5852 PMC7254182

[55] Lagisetty PA, Ross R, Bohnert A, et al. Buprenorphine treatment divide by race/ethnicity and payment. JAMA Psychiatry. 2019;76(9):979–981. 31066881 10.1001/jamapsychiatry.2019.0876 PMC6506898

[56] Larochelle MR, Slavova S, Root ED, et al. Disparities in opioid overdose death trends by race/ethnicity, 2018-2019, from the HEALing communities study. Am J Public Health. 2021;111(10):1851–1854. 34499540 10.2105/AJPH.2021.306431 PMC8561170

[57] Manhapra A, Quinones L, Rosenheck R. Characteristics of veterans receiving buprenorphine vs. methadone for opioid use disorder nationally in the Veterans Health Administration. Drug Alcohol Depend. 2016;160:82–89. 26804898 10.1016/j.drugalcdep.2015.12.035 PMC4767635

[58] Mason M, Soliman R, Kim HS, et al. Disparities by sex and race and ethnicity in death rates due to opioid overdose among adults 55 years or older, 1999 to 2019. JAMA Netw Open. 2022;5(1):e2142982. 35015062 10.1001/jamanetworkopen.2021.42982 PMC8753495

[59] Iyengar S, Kravietz A, Bartholomew TS, et al. Baseline differences in characteristics and risk behaviors among people who inject drugs by syringe exchange program modality: an analysis of the Miami IDEA syringe exchange. Harm Reduct J. 2019;16(1):7. 30674334 10.1186/s12954-019-0280-z PMC6343273

[60] Hall G, Neighbors CJ, Iheoma J, et al. Mobile opioid agonist treatment and public funding expands treatment for disenfranchised opioid-dependent individuals. J Subst Abuse Treat. 2014;46(4):511–515. 24468235 10.1016/j.jsat.2013.11.002

[61] Practice guidelines for the administration of buprenorphine for treating opioid use disorder. Health and Human Services Department; 2021.

[62] Bouza E, Kestler M, Beca T, et al. The NOVA score: a proposal to reduce the need for transesophageal echocardiography in patients with enterococcal bacteremia. Clin Infect Dis. 2015;60(4):528–535. 25381321 10.1093/cid/ciu872

[63] Carrico AW, et al. Randomized controlled trial of a positive affect intervention to reduce HIV viral load among sexual minority men who use methamphetamine. J Int AIDS Soc. 2019;22(12):e25436. 31860172 10.1002/jia2.25436 PMC6924317

[64] Ahmadi J, Jahromi MS, Ghahremani D, et al. Single high-dose buprenorphine for opioid craving during withdrawal. Trials. 2018;19(1):675. 30526648 10.1186/s13063-018-3055-z PMC6288888

[65] Ang-Lee K, Oreskovich MR, Saxon AJ, et al. Single dose of 24 milligrams of buprenorphine for heroin detoxification: an open-label study of five inpatients. J Psychoactive Drugs. 2006;38(4):505–512. 17373566 10.1080/02791072.2006.10400589

[66] Ciccarone D. Editorial for “US heroin in transition: supply changes, fentanyl adulteration and consequences” IJDP special section. Int J Drug Policy. 2017;46:107–111. 28735776 10.1016/j.drugpo.2017.06.010 PMC5742018

[67] Ciccarone D. The triple wave epidemic: supply and demand drivers of the US opioid overdose crisis. Int J Drug Policy. 2019;71:183–188. 30718120 10.1016/j.drugpo.2019.01.010 PMC6675668

[68] Herring AA, Vosooghi AA, Luftig J, et al. High-dose buprenorphine induction in the emergency department for treatment of opioid use disorder. JAMA Netw Open. 2021;4(7):e2117128. 34264326 10.1001/jamanetworkopen.2021.17128 PMC8283555

[69] Volkow ND. The epidemic of fentanyl misuse and overdoses: challenges and strategies. World Psychiatry. 2021;20(2):195–196. 34002497 10.1002/wps.20846 PMC8129846

[70] Chan B, Gean E, Arkhipova-Jenkins I, et al. Retention strategies for medications for opioid use disorder in adults: a rapid evidence review. J Addict Med. 2021;15(1):74–84. 32956162 10.1097/ADM.0000000000000739 PMC7864607

[71] Hochheimer M, Unick GJ. Systematic review and meta-analysis of retention in treatment using medications for opioid use disorder by medication, race/ethnicity, and gender in the United States. Addict Behav. 2022;124:107113. 34543869 10.1016/j.addbeh.2021.107113

[72] Kennedy AJ, Wessel CB, Levine R, et al. Factors associated with long-term retention in buprenorphine-based addiction treatment programs: a systematic review. J Gen Intern Med. 2022;37(2):332–340. 33469778 10.1007/s11606-020-06448-z PMC8810983

[73] Timko C, Schultz NR, Cucciare MA, et al. Retention in medication-assisted treatment for opiate dependence: a systematic review. J Addict Dis. 2016;35(1):22–35. 26467975 10.1080/10550887.2016.1100960 PMC6542472

[74] Carroll KM, Weiss RD. The role of behavioral interventions in buprenorphine maintenance treatment: a review. Am J Psychiatry. 2017;174(8):738–747. 27978771 10.1176/appi.ajp.2016.16070792 PMC5474206

[75] Feelemyer J, Des Jarlais D, Arasteh K, et al. Retention of participants in medication-assisted programs in low- and middle-income countries: an international systematic review. Addiction. 2014;109(1):20–32. 23859638 10.1111/add.12303 PMC5312702

[76] McLaughlin MF, Li R, Carrero ND, et al. Opioid use disorder treatment for people experiencing homelessness: a scoping review. Drug Alcohol Depend. 2021;224:108717. 33985863 10.1016/j.drugalcdep.2021.108717 PMC9758007

[77] Wyse JJ, Morasco BJ, Dougherty J, et al. Adjunct interventions to standard medical management of buprenorphine in outpatient settings: a systematic review of the evidence. Drug Alcohol Depend. 2021;228:108923. 34508958 10.1016/j.drugalcdep.2021.108923 PMC9063385

[78] Becker SJ, Scott K, Helseth SA, et al. Effectiveness of medication for opioid use disorders in transition-age youth: a systematic review. J Subst Abuse Treat. 2022;132:108494. 34098208 10.1016/j.jsat.2021.108494 PMC8628023

[79] Fareed A, Vayalapalli S, Casarella J, et al. Effect of buprenorphine dose on treatment outcome. J Addict Dis. 2012;31(1):8–18. 22356665 10.1080/10550887.2011.642758

[80] McCarty D, Chan B, Buchheit BM, et al. Effectiveness of and access to medications for opioid use disorder for adolescents and young adults: a scoping review. J Addict Med. 2022;16(3):e157–e164. 34282085 10.1097/ADM.0000000000000898 PMC8761222

[81] Viera A, Bromberg DJ, Whittaker S, et al. Adherence to and retention in medications for opioid use disorder among adolescents and young adults. Epidemiol Rev. 2020;42(1):41–56. 32239206 10.1093/epirev/mxaa001 PMC8087870

[82] Malta M, Varatharajan T, Russell C, et al. Opioid-related treatment, interventions, and outcomes among incarcerated persons: a systematic review. PLoS Med. 2019;16(12):e1003002. 31891578 10.1371/journal.pmed.1003002 PMC6938347

[83] Bachhuber MA, Thompson C, Prybylowski A, et al. Description and outcomes of a buprenorphine maintenance treatment program integrated within prevention point philadelphia, an urban syringe exchange program. Subst Abus. 2018;39(2):167–172. 29474119 10.1080/08897077.2018.1443541 PMC9333078

[84] Hill K, Nussdorf L, Mount JD, et al. Initiation of low-threshold buprenorphine in nontreatment seeking patients with opioid use disorder engaged in hepatitis C treatment. J Addict Med. 2022;16(1):10–17. 33560694 10.1097/ADM.0000000000000807 PMC8923533

[85] Hood JE, Banta-Green CJ, Duchin JS, et al. Engaging an unstably housed population with low-barrier buprenorphine treatment at a syringe services program: lessons learned from Seattle, Washington. Subst Abus. 2020;41(3):356–364. 31403907 10.1080/08897077.2019.1635557

[86] Darke S. Self-report among injecting drug users: a review. Drug Alcohol Depend. 1998;51(3):253–263. 9787998 10.1016/s0376-8716(98)00028-3

